# Genetic Diversity and Expanded Host Range of J Paramyxovirus Detected in Wild Small Mammals in China

**DOI:** 10.3390/v15010049

**Published:** 2022-12-23

**Authors:** Yunfa Zhang, Jingtao Zhang, Yuna Wang, Feng Tian, Xiaolong Zhang, Gang Wang, Shuang Li, Heng Ding, Zhenyu Hu, Wei Liu, Xiaoai Zhang

**Affiliations:** 1 State Key Laboratory of Pathogen and Biosecurity, Beijing Institute of Microbiology and Epidemiology, Beijing 100071, China; 2 Urumqi Customs Port Outpatient Department, Xinjiang International Travel Health Care Center, Urumqi 830011, China; 3 Science and Technology Research Center of China Customs (STRC), Beijing 100026, China; 4 School of Public Health, Anhui Medical University, Hefei 230032, China

**Keywords:** paramyxoviruses, J paramyxovirus, *Jeilongvirus*, mammals

## Abstract

J paramyxovirus (JPV) is a rodent-borne Jeilongvirus isolated from moribund mice (*Mus musculus*) with hemorrhagic lung lesions trapped in the 1972 in northern Queensland, Australia. The JPV antibodies have been detected in wild mice, wild rats, pigs, and human populations in Australia. Here, by next-generation sequencing (NGS), we detected JPV from *M. musculus* in Shandong Province of China. Molecular detection of JPV was performed to survey to survey the infection among 66 species of wild small mammals collected from six eco-climate regions in China by applying JPV specific RT-PCR and sequencing. Altogether, 21 out of 3070 (0.68%) wild small mammals of four species were positive for JPV, including 5.26% (1/19) of *Microtus fortis*, 3.76% (17/452) of *M. musculus*, 1.67% (1/60) of *Apodemus peninsulae*, and 0.48% (2/421) of *Apodemus agrarius*, which captured three eco-climate regions of China (northeastern China, northern China, and Inner Mongolia-Xinjiang). Sequence analysis revealed the currently identified JPV was clustered with other 14 Jeilongvirus members, and shared 80.2% and 89.2% identity with Australia’s JPV partial RNA polymerase (L) and glycoprotein (G) genes, respectively. Phylogenetic analysis demonstrated the separation of three lineages of the current JPV sequences. Our results show three new hosts (*A. agrarius*, *A. peninsulae*, and *M. fortis*) for JPV, most of which were widely distributed in China, and highlight the potential zoonotic transmission of JPV in humans. The detection of JPV in wild small mammals in China broaden the viral diversity, geographical distribution, and reservoir types of JPV. Future studies should prioritize determining the epidemiological characteristics of JPV, so that potential risks can be mitigated.

## 1. Introduction

The *Paramyxoviridae* family currently comprises RNA viruses of four subfamilies, 17 genera, and 86 species (https://talk.ictvonline.org/, accessed on 15 November 2022), many with high zoonotic potential. The members of the *Paramyxoviridae* family are negative-sense single-stranded RNA viruses, and some of them are capable of infecting a wide range of natural hosts, from reptiles, birds, and fish to a variety of mammals, including humans [1]. Of special interest is the newly proposed genus *Jeilongvirus*, in the sub-family *Orthoparamyxovirinae,* to which the *Henipavirus*, *Morbillivirus,* and *Respirovirus* genera belong [2,3]. Several unclassified rodent-borne and bat-borne viruses that have emerged in the past decade have been classified to this newly established genus, attaining a total of 15 paramyxoviruses species, including eight rodent-borne viruses (J paramyxovirus, JPV; Beilong virus, BeiV; rodent paramyxovirus, RoPV; ruloma virus, RulV; Mount Mabu Lophuromys virus 1, MMLV-1; Mount Mabu Lophuromys virus 2, MMLV-2; Pohorje Myodes paramyxovirus 1, PMPV-1; Tailam virus, TaiV), five bat-borne viruses (Miniopterus schreibersii paramyxovirus, MisPV; bat Paramyxovirus 16797, BatPV-1; bat paramyxovirus 17770, BatPV-2; bat paramyxovirus, BatPV-3; Shaan virus, ShaV), belerina virus (BeV) from erinaceus, and feline paramyxovirus (FPaV) from felis catus, demonstrating the vast host range of Jeilongviruses [4,5,6,7,8,9]. Although the *Paramyxoviridae* family of viruses has been previously recognized as biomedically and veterinarily important, the recently emerging Jeilongviruses have been rarely investigated, particularly for JPV. JPV was first isolated in 1972 from moribund mice (*Mus musculus*) trapped in northern Queensland, Australia, by using kidney autoculture [10]. Extensive hemorrhagic lung lesions were shown in the mice from which the virus was isolated, indicating pathogenicityin rodent hosts [10]. In the subsequent study performed in Australia, JPV-LW (named after Lin-Fa Wang, who determined its sequence) that did not cause any disease in JPV-LW-infected mice was determined [11]. JPV antibodies have been detected in wild mice, wild rats, pigs, and human populations in Australia [10], suggesting a wide host range of JPV. Our previous study had confirmed the existence of BeiV in a high variety of wild animals across six eco-climate regions in China [12]. The current study was designed to determine the molecular evidence of JPV in a great variety of wild small mammals in a wide region of China.

## 2. Materials and Methods

### 2.1. Sample Collection

Wild small mammals were captured with snap traps from 2013 to 2021 in 11 provinces, autonomous regions, and municipalities in China, i.e., Shandong, Guangdong, Zhejiang, Liaoning, Henan, Jilin, Heilongjiang, Yunnan, Xinjiang, Inner Mongolia, and Beijing. The spleen samples were collected and kept at −80 °C. The captured animals were morphologically identified, and confirmation of species identification was performed by amplifying the mitochondrial cytochrome *b* (*mt-cyt b*) gene region and determining the DNA sequence [13].

### 2.2. Next-Generation Sequencing (NGS)

Twelve randomly selected spleen samples from four dominant wild small animal species (*M. musculus*, *Apodemus agrarius*, *Rattus norvegicus*, and *Tscherskia triton*) in Shandong Province were subject to metagenomic analysis by NGS [14]. Briefly, total RNA was extracted using the AllPrep DNA/RNA Mini Kit (Qiagen, Hilden, Germany), from which rRNA was removed using the MGIEasy rRNA Depletion Kit (BGI, China). A high-throughput sequencing library was constructed using an MGIEasy RNA Library Prep Kit (BGI). Viral gene libraries were then sequenced using the MGI2000 platform (BGI), sequencer with pair-end (150-bp) reads. After processing the original data by filtering, trimming, and error removal, the remaining reads were mapped to NCBI viral reference genome sequences using Bowtie2 (version: 2.3.5.1) [15]. De novo assembly was performed using MEGAHIT (v1.2.9) software [16]. The assembled contigs were compared against the NCBI non-redundant nucleotide database using Blastn (version 2.12.0+) [17].

### 2.3. Reverse Transcription-PCR (RT-PCR) Sequencing for JPV

Total nucleic acid was extracted using the AllPrep DNA/RNA Mini Kit (Qiagen) from spleen samples. The JPV screening was performed by PCR amplification of a 199-bp fragment of the glycoprotein (G) gene using JPV- specific primers (JPVF 5′- AAAACTTAGGAGTGAATGAGCGTC -3′ and JPVR 5′- TGGTAAATAAGCTCAACAGTCCAG -3′) designed based on genomic sequences obtained by metagenomic analyses and all nucleotide sequences from the currently determined JPV. RT-PCR amplification was performed using the PrimeScript™ One Step RT-PCR kit. RT-PCR conditions were identical to those described previously except for an annealing temperature of 55 °C for JPV. The 199-bp amplicon was separated on a 3% agarose gel. PCR products from positive samples were used to determine sequences with an applied biosystem (ABI 3730XL DNA sequencer). All PCR tests were conducted in parallel with the positive control (RNA from the positive sample) and the negative control (RNase-free water).

### 2.4. Phylogenetic Analysis

The nucleotide and amino acid sequences from the currently determined JPV and representative species belonging to the family *Phenuiviridae* that were downloaded from GenBank (Table S1) were aligned by the ClustalW method using MEGA-X [18]. Phylogenetic trees were constructed with MEGA-X by the maximum likelihood (ML) method with 1000 bootstrap replications. Based on the Bayesian information criterion (BIC), the best evolutionary model was selected to run the analysis and trees were generated using the general time reversible model.

### 2.5. Statistical Analysis

The chi-square or Fisher exact test was performed to compare the detection rate of JPV regarding the geographic region. Statistical analyses were performed using R (version 3.5.3). All statistical tests were 2-tailed, and a significance level (*P*) of 0.05 was used.

### 2.6. Nucleotide Sequence Accession Numbers

The JPV partial sequences generated in this study were submitted to GenBank under the accession numbers OP795118-OP795139.

## 3. Results

### 3.1. Study Site and Sample Collection

**Figure 1:**
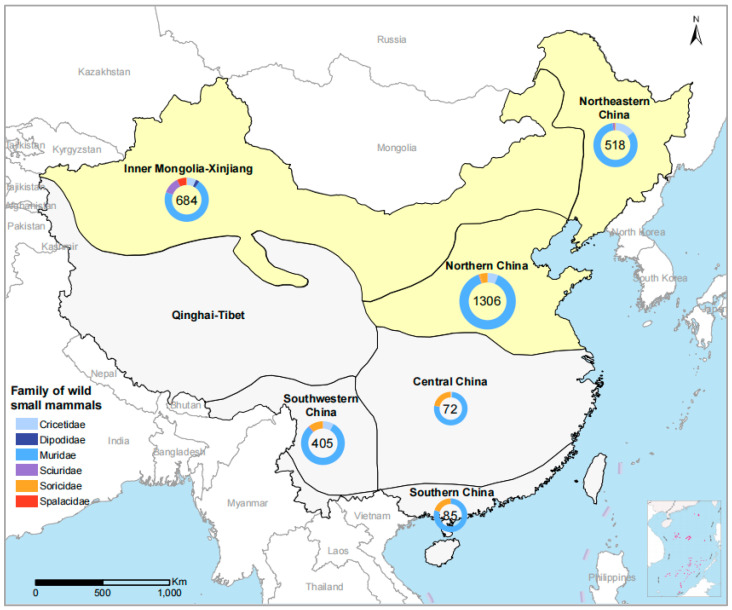
Map of China showing the collection sites for wild small mammals for JPV detection. The distribution map was generated by Arcgis 10.2. Wild animal sampling was indicated in pies. The sampling number for each region was marked inside. The proportion of families wild of animals is shown by different colors in the pie charts. The JPV positive ecoclimate regions are shown in yellow.

A total of 3070 wild small mammals that belonged to 66 species in six families were trapped, including 2518 from 29 species of the Muridae family, 239 from 15 species of the *Cricetidae* family, 148 from 12 species of the *Soricidae* family, 100 from 6 species of *Sciuridae* family, 47 from 2 species of *Spalacidae* family, and 18 from 2 species of the *Dipodidae* family (Figure 1, Table S2). The 11 surveyed locations were grouped into six eco-climate regions [19]. Eco-climate regions were used for the geographic description. The highest number of samples were collected from northern China (1306/3070, 42.54%), followed by Inner Mongolia-Xinjiang (684/3070, 22.28%), northeastern China (518/3070, 16.87%), southwestern China (405/3070, 13.19%), southern China (85/3070, 2.77%), and central China (72/3070, 2.35%) (Table 1, Figure 1).

### 3.2. Identification of JPV in M. musculus by NGS

Of four dominant wild small animal species subject to metagenomic analysis, JPV specific sequences were identified from spleen samples of *M. musculus* captured in Shandong Province in 2018. Out of a total of 1142 reads obtained, 794 (69.5%) were annotated to JPV. No JPV-specific sequence was obtained from the other three dominant wild small animal species (*A*. *agrarius*, *R*. *norvegicus*, and *T*. *triton*) in Shandong Province by NGS.

**Figure 2:**
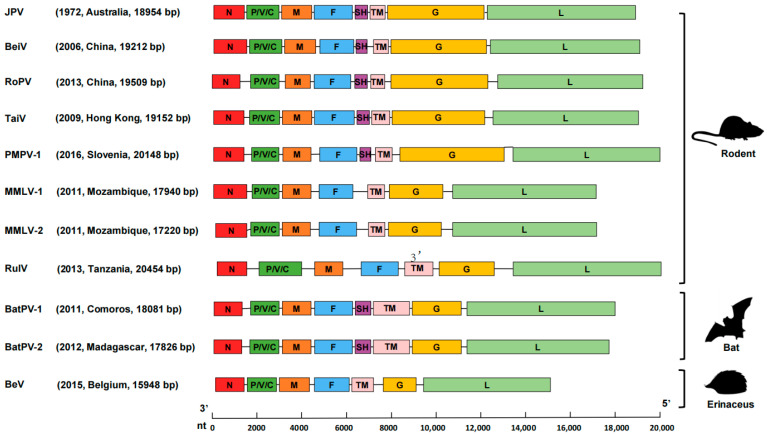
Genome organization of JPV and representative members of the genus *Jeilongvirus*. The diagram is drawn to scale, and the scale bar is shown at the bottom. The abbreviation nt denotes nucleotides. J paramyxovirus, JPV; Beilong virus, BeiV; rodent paramyxovirus, RoPV; Tailam virus, TaiV; Pohorje Myodes paramyxovirus 1, PMPV-1; Mount Mabu Lophuromys virus 1, MMLV-1; Mount Mabu Lophuromys virus 2, MMLV-2; ruloma virus, RulV; bat Paramyxovirus 16797, BatPV-1; bat paramyxovirus 17770, BatPV-2; belerina virus, BeV. The genomes of Jeilongviruses range from 15,948 to 20,148 nucleotides (nt) in length, which encode nucleocapsid (N), phosphoprotein (P), matrix protein (M), fusion protein (F), small hydrophobic protein (SH), transmembrane protein (TM), glycoprotein (G), and RNA polymerase (L).

**Figure 3:**
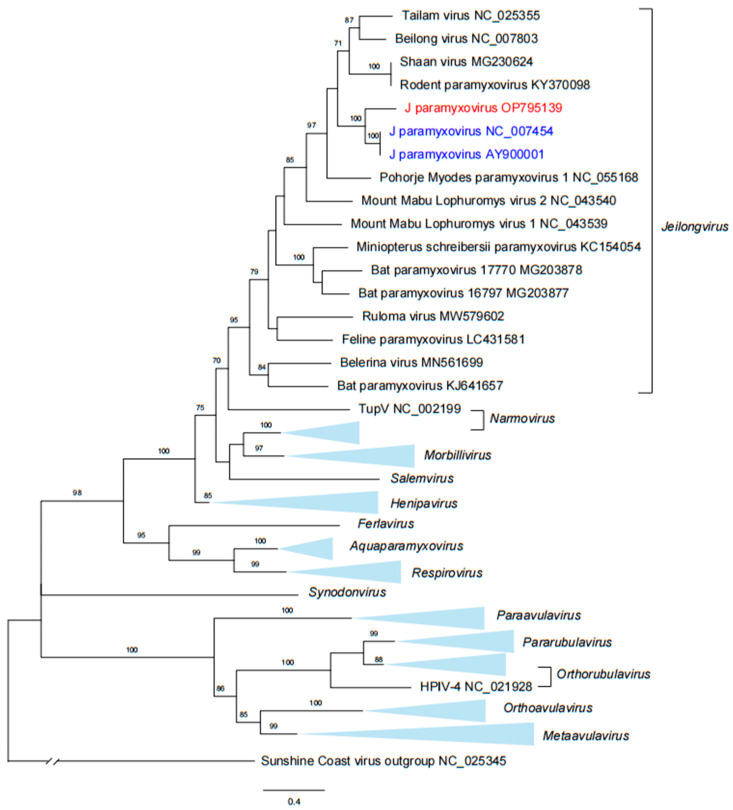
Phylogenetic analysis of the L segment of the JPV. The phylogenetic tree was constructed based on the 709-bp fragment of partial L segment. The Australian JPV sequences were labeled in blue. The current JPV sequence was labeled in red. Trees were generated using MEGA-X and analyzed included 1000 bootstrap replicates. J paramyxovirus, JPV.

The nucleotide sequences of the current JPV (strain SD634) share 80.2% and 89.2% identity for partial RNA polymerase (L) and G genes, respectively, with Australian JPVs that were isolated from feral rodents in 1972 (GenBank accession numbers NC007454 and AY900001). The genome of JPV is composed of 18,954 nucleotides with a genome organization similar to that of other Jeilongviruses (Figure 2). Phylogenetic trees based on a 709-bp fragment of the L gene indicated that the current JPV strain SD634 grouped with previously described Australian JPVs, showing a close relationship with the other 14 species in the genus *Jeilongvirus* (Figure 1)*,* while being distinct from other members of the *Paramyxoviridae* family (Figure 3). The alignment with the other 14 species in the genus *Jeilongvirus* showed similarity of 73.2% (for ShaV and RoPV), 71.7% (TaiV), 70.8% (PMPV-1), 70.6% (BeiV), 68.6% (MMLV-2), 67.2% (FPaV), 65.5% (MisPV), 65.0% (MMLV-1), 64.8% (BatPV-1), 63.4% (BatPV-2), 62.9% (BeV), 62.4% (BatPV-3), and 62.2% (RulV) (Table S2).

### 3.3. JPV Screening in Wild Small Mammals by RT-PCR

All specimens (*n* = 3070) were individually screened for JPV by RT-PCR using a self-designed primer targeting the JPV G gene. Altogether, 21 out of 3070 (0.68%) mammals of four species were tested positive for JPV (Table 1), including *M. musculus* (house mouse, 17/452), *A. agrarius* (striped field mouse, 2/421), *Apodemus peninsulae* (Korean field mouse, 1/60) and *Microtus fortis* (reed vole, 1/19) (Table 1, Table S3). The positive samples spanned a 4-year period from 2017 to2021.

Positive results were originated from three of the six sampled eco-climate regions, including northern China (1.15%, 15/1306), Inner Mongolia-Xinjiang (0.58%, 4/684), and northeastern China (0.39%, 2/518) (Table 1). Although the positive rates did not differ significantly among the six sampled ecoclimate regions (*p* = 0.118), the highest frequency observed in northern China compared to the other five sampled ecoclimate regions (*p* = 0.014). All positive JPV detection in Inner Mongolia-Xinjiang was from *M. Musculus* (4.49%, 4/89). Three species of wild small mammals yielded positive detection in northern China, i.e., *M. musculus* of the genus *Mus* (3.72%, 13/349), *A. agrarius* (0.45%, 1/220), and *A. peninsulae* (2.50%, 1/40) of the genus *Apodemus*. Two species of wild small mammals with positive JPV detection were determined in northeastern China, *A. agrarius* (0.59%, 1/170) and *M. forti* (5.56%, 1/18) of the genus *Microtus*, respectively. No positive detection was obtained from the 38 species of wild small mammals in the other three ecoclimate regions, probably due to the small sample size.

### 3.4. Phylogenetic Analysis of JPV Sequences

**Figure 4:**
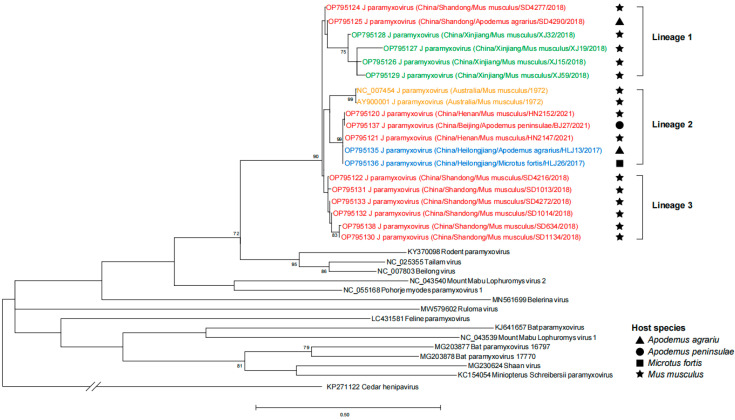
Phylogenetic analysis of partial G segment of JPV. The phylogenetic tree constructed based on partial nucleotide sequences of G gene from 21 JPV-positive samples. The Australian JPV sequences were labeled in orange. The current JPV were labeled in red (northern China), blue (northeastern China) and green (Inner Mongolia-Xinjiang). Trees were generated using MEGA-X and analyzed included 1000 bootstrap replicates. J paramyxovirus, JPV.

For each of the 21 JPV-positive animals, partial sequences (199 bp) of the G gene were obtained for the phylogenetic analyses, together with sequences of 14 Jeilongvirus members retrieved from GenBank. Three major lineages were formed, which showed a close relatedness between the viral clades and their ecoclimate regions. For instance, strains from Inner Mongolia-Xinjiang were clustered into Lineage 1, strains from northeastern China were clustered into Lineage 2, and sequences from northern China were observed in all three lineages. No host specificity can be observed, since *M. musculus* can harbor all three lineages, and *A. agrarius* can harbor both Lineages 1 and 2. (Figure 4). The Australian JPV was clustered in Lineage 2, together with the current JPV from both northern and northeastern China (Figure 4).

## 4. Discussion

The *Paramyxoviridae* family contains several zoonotic viruses, including highly pathogenic viruses, such as Nipah virus and Hendra virus, and an increasing number of largely uncharacterized animal viruses [20,21,22]. After its first emergence in Australia in 1972, no distribution in other countries was reported. Here, we determined JPV in three other rodent species than the already known one in Australia for the first time, indicating a diverse geographical range and a over long period during which JPV can circulate in China.

Recently, new Jeilongviruses have emerged from an extensive range of small mammals, however, very little is known about the distribution and diversity of the rapidly expanding genus *Jeilongvirus*. Among this variety of Jeilongvirus genus, BeiV and JPV have been highlighted with the potential for zoonotic spread to humans. Serological evidence suggests JPV has previously spilled over into the human population, although only limited to Australia [10], while BeiV was capable of cross-contaminating human cell cultures from rodent cell cultures [5]. JPV and BeiV have similar genome organizations and interchangeable genome replication machines. In contrast to most other paramyxoviruses, the JPV and BeiV V proteins did not interact with or inhibit signaling by STAT1 or STAT2; however, V proteins of both viruses interacted with melanoma differentiation-associated protein 5 (MDA5) and inhibited MDA5-dependent activation of the IFN-β promoter in HEK293T cells [23].

While both belong to the *Jeilongvirus* genus of the *Paramyxoviridae* family, JPV appeared to have a lower prevalence, host diversity, and a narrower geographic range as compared to BeiV. BeiV was discovered in a human kidney cell line in 2006 in Hong Kong, China [5], and *R. norvegicus* and *R. rattus* were suggested as natural reservoirs of BeiV by phylogenetic analysis of the naturally occurring BeiV [24]. Subsequent epidemiological studies from our group have determined the presence of BeiV in a total of 22 species of wild rodents and shrews in China, with *R. norvegicus* and *R. rattus* as the most predominant species that carry the virus [12]. In this study, the same batch of wild small mammals was tested for the presence of JPV, and thus, provided comparisons for the distribution and hosts of JPV and BeiV. It was determined that the three ecoclimate regions (northeastern China, Inner Mongolia-Xinjiang, and northern China) identified both viruses, and three rodent hosts (*A. agrarius*, *A. peninsulae*, and *M. musculus*) harbored both viruses. No codetection in one single sample was determined. After its first emergence in Australia in 1972, little is known about the ecology of JPV in other parts of the world. In this study, in addition to *M. musculus*, our results show three new potential hosts, *A. agrarius*, *A. peninsulae*, and *M. fortis* for JPV, most of which were widely distributed in China and wide range and highlight potential zoonotic transmission of JPV in humans. A wide screen for JPV is needed in regions where these potential hosts are highly abundant. Serological tests in wildlife and humans should also be carried out to extend the knowledge on its potential importance in causing disease.

In this study, although JPV was mainly tested in *M. musculus* from three ecoclimate regions, *M. musculus* is extensively distributed in China. It is plausible to assume that both ecological climates and topographic features as well as the ecological characteristics of animals, may play a role in the natural circulation of JPV. However, these results warrant further confirmation in future studies.

In conclusion, we determined the presence of JPV in four rodent species in China, also representing its first identification outside Australia. Although with low prevalence, these results expand on the known diversity of rodent species that can host JPV and their geographical distribution in the worldwide range. Further studies that combine active surveillance, viral isolation, and a phylogenetic approach might help to gain better knowledge on this scarcely investigated virus and guide the diagnosis and management of its potential infection in human beings.

## Figures and Tables

**Table 1:** Wild small mammals screened for J paramyxovirus in China.

Eco-Climate Regions	Year	Species (No. of Positive/No. Tested)	No. Total Tested	No. (%) of Positive
**Northern**			1306	15 (1.15)
Shandong	2018	*Apodemus agrarius* (1/115), *Mus musculus* (9/286), *Rattus norvegicus* (0/134), *Tscherskia triton* (0/42), *Suncus murinus* (0/13), *Crocidura lasiura* (0/20), *Crocidura shantungensis* (0/7) and *Crocidura tanakae* (0/15)	632	10 (1.58)
Henan	2018, 2021	*Apodemus agrarius* (0/54), *Apodemus draco* (0/4), *Callosciurus erythraeus* (0/5), *Crocidura tanakae* (0/6), *Episoriculus fumidus* (0/2), *Mus musculus* (4/60), *Niviventer andersoni* (0/2), *Niviventer confucianus* (0/5), *Niviventer niviventer* (0/18), *Rattus norvegicus* (0/24), *Rattus tanezumi* (0/285) and *Suncus murinus* (0/1)	466	4 (0.86)
Beijing	2018	*Allocricetulus eversmanni* (0/6), *Apodemus agrarius* (0/51), *Apodemus draco* (0/56), *Apodemus peninsulae* (1/40), *Cricetulus longicaudatus* (0/28), *Mus musculus* (0/3), *Myodes rufocanus* (0/3), *Niviventer confucianus* (0/19), *Suncus murinus* (0/1) and *Tscherskia triton* (0/1)	208	1 (0.48)
**Inner Mongolia-Xinjiang**			684	4 (0.58)
Inner Mongolia	2018, 2019, 2021	*Allactaga sibirica* (0/11), *Cricetulus barabensis* (0/2), *Cricetulus migratorius* (0/37), *Dipus sagitta* (0/5), *Meriones meridianus* (0/57), *Meriones unguiculatus* (0/181), *Mus musculus* (0/4), *Myospalax aspalax* (0/2), *Myospalax psilurus* (0/45), *Phodopus roborovskii* (0/4), *Rattus pyctoris* (0/1) and *Spermophilus dauricus* (0/28)	377	0 (0)
Xinjiang	2017–2019	*Meriones libycus* (0/42), *Rhombomys opimus* (0/74), *Apodemus sylvaticus* (0/15), *Mus musculus* (4/89), *Spermophilus undulatus* (0/25), *Suncus murinus* (0/2), *Microtus oeconomus* (0/1), *Cricetulyus migratorius* (0/4), *Rattus norvegicus* (0/20), *Spermophilus erythrogenys* (0/31), *Allactaga sibirica* (0/2) and *Meriones tamariscinus* (0/2)	307	4 (1.30)
**Northeastern**			518	2 (0.39)
Heilongjiang	2017, 2021	*Apodemus agrarius* (1/170), *Apodemus peninsulae* (0/12), *Microtus fortis* (1/18), *Microtus maximowiczii* (0/1), *Mus musculus* (0/2), *Myodes rufocanus* (0/4), *Myodes rutilus* (0/5), *Rattus norvegicus* (0/110), *Tamias sibiricus* (0/7) and *Tscherskia triton* (0/1)	330	2 (0.61)
Jilin	2017	*Apodemus peninsulae* (0/8), *Crocidura tanakae* (0/2), *Myodes rufocanus* (0/1), *Myodes rutilus* (0/47), *Sorex isodon* (0/1) and *Tamias sibiricus* (0/1)	60	0 (0)
Liaoning	2017, 2018	*Mus musculus* (0/8) and *Rattus norvegicus* (0/120)	128	0 (0)
**Southwestern**			405	0 (0)
Yunnan	2013, 2015, 2016	*Suncus murinus* (0/4), *Rattus tanezumi* (0/144), *Crocidura tanakae* (0/5), *Crocidura lasiura* (0/4), *Rattus yunnanensis* (0/9), *Rattus steini* (0/7), *Tamiops swinhoei* (0/3), *Niviventer coxingi* (0/7), *Apodemus ilex* (0/80), *Anourosorex squamipes* (0/15), *Eothenomys eleusis* (0/8), *Sorex bedfordiae* (0/8), *Blarinella quadraticauda* (0/4), *Niviventer andersoni* (0/4), *Episoriculus caudatus* (0/3), *Melomys burtoni* (0/2), *Rattus brunneusculus* (0/7), *Berylmys bowersi* (0/1), *Mus pahari* (0/6), *Apodemus chevrieri* (0/56), *Eothenomys cachinus* (0/1), *Crocidura horsfieldii* (0/1), *Chiropodomys gliroides* (0/1), *Micromys minutus* (0/1), *Eothenomys miletus* (0/16) and *Eothenomys proditor* (0/8)	405	0 (0)
**Central**			72	0 (0)
Zhejiang	2018, 2012	*Apodemus agrarius* (0/31), *Crocidura lasiura* (0/9), *Crocidura shantungensis* (0/5), *Microtus fortis* (0/1), *Niviventer confucianus* (0/9), *Niviventer fulvescens* (0/15) and *Sorex caecutiens* (0/2)	72	0 (0)
**Southern**			85	0 (0)
Guangdong	2017, 2021	*Bandicota indica* (0/14), *Crocidura tanakae* (0/3), *Rattus andamanensis* (0/11), *Rattus norvegicus* (0/38), *Rattus tanezumi* (0/5) and *Suncus murinus* (0/14)	85	0 (0)
**Total**			3070	21 (0.68)

## Data Availability

Not applicable.

## Supplementary Materials

The following supporting information can be downloaded at: https://www.mdpi.com/article/10.3390/v15010049/s1, Table S1: GenBank accession numbers of viruses used for phylogenetic analysis in this study; Table S2: Nucleotide similarity (%) between our J paramyxovirus and other 14 species in genus *Jeilongvirus* based on a 709-bp fragment of the L gene; Table S3: Positive rate of J paramyxovirus in wild small mammals in six eco-climate regions in China.
