# Supplementary material for: Genetic Diversity and Expanded Host Range of J Paramyxovirus Detected in Wild Small Mammals in China
Source: Viruses. 2022 Dec 23;15(1):49. doi: 10.3390/v15010049 (PMC9866557; doi:10.3390/v15010049)
Supplement: Supplementary file 1 [file viruses-15-00049-s001.zip › viruses-2071232-supplementary.pdf]

Table S1. GenBank accession numbers of viruses used for phylogenetic analysis in this study.

Genus	Species	Abbreviation	GenBank Accession Number (Nucleotide)	GenBank Accession Number (Amino Acid)
Metaavulavirus	Avian metaavulavirus 2	APMV-2	HM159993.1	ACA49110.1
	Avian metaavulavirus 5	APMV-5	GU206351.1	ADD39006.1
	Avian metaavulavirus 6	APMV-6	AY029299.1	ABQ45549.1
	Avian metaavulavirus 7	APMV-7	FJ231524.1	ACN72645.1
	Avian metaavulavirus 8	APMV-8	FJ619036.1	AUJ87608.1
	Avian metaavulavirus 10	APMV-10	HM147142.3	ADK12969.2
	Avian metaavulavirus 11	APMV-11	JQ886184.1	AFN06859.1
	Avian metaavulavirus 14	APMV-14	KX258200.1	APP90895.1
	Avian metaavulavirus 15	APMV-15	KX932454.2	ARO49358.2
	Avian metaavulavirus 20	APMV-20	MF033136.1	ACO48302.2
Orthoavulavirus	Avian orthoavulavirus 1	APMV-1	AF309418.1	AAS67167.1
	Avian orthoavulavirus 9	APMV-9	EU910942.1	ACJ82944.1
	Avian orthoavulavirus 12	APMV-12	KC333050.1	AGH32603.1
	Avian orthoavulavirus 13	APMV-13	MN150295	BAV03985.1
	Avian orthoavulavirus 16	APMV-16	KY511044.1	ARV85980.1
	Avian orthoavulavirus 17	APV-B(17)	KY452442.1	ARU83011.1
	Avian orthoavulavirus 18	APV-B(18)	KY452443.1	ARU83017.1
	Avian orthoavulavirus 19	APV-B(19)	KY452444.1	ARU83023.1
	Avian orthoavulavirus 21	APMV-21	MF594598.1	-
Paraavulavirus	Avian paraavulavirus 3	APMV-3	EU782025.1	ACI47553.1
	Avian paraavulavirus 4	APMV-4	JX987283.1	ACF60579.1
Synodonvirus	Synodus synodonvirus	WTLPV	MG600058	AVM87369.1
Aquaparamyxovirus	Oncorhynchus aquaparamyxovirus	PSPV	MH900517	AYN62580.1
	Salmo aquaparamyxovirus	SAV	EU156171.1	ABX57743.1
Ferlavirus	Reptilian ferlavirus	FDLV	AY141760.2	AAN18266.1
Henipavirus	Nipah henipavirus	NiV	AF212302.2	AAK29089.1
	Hendra henipavirus	HeV	AF017149.3--ok	AAC83194.3
	Hendra henipavirus genotype 2	HeV-g2	MZ229746	-
	Cedar henipavirus	CedV	JQ001776.1	AFP87280.1
	Ghanaian bat henipavirus	GhV	HQ660129.1	AET43339.1
	Mojiang henipavirus	MojV	KF278639.1	AHM23778.1
	Daeryong virus	DARV	MZ574408	-
	Gamak virus	GAKV	MZ574409	-
Jeilongvirus	Beilong jeilong virus	BeiV	NC007803	AAZ82812.1
	Jun jeilongvirus	JPV	AY900001	AAX86035.1
	Jun jeilongvirus	JPV	NC007454	YP_338085.1
	Lophuromys jeilongvirus 1	MMLV-1	MG573140	AVM86019.1

	Jeilongvirus anhuiense	MisPV	KC154054	AGU69458.1
	Jeilongvirus apodemi	RoPV	KY370098	ATP66853.1
	Jeilongvirus comorosense	BatPV-1	MG203877	AYM47528.1
	Jeilongvirus erinacei	BeV	MN561699	QKZ93219.1
	Jeilongvirus felis	FPaV	LC431581	BBG92175.1
	Jeilongvirus madagascarense	BatPV-2	MG203878	AYM47538.1
	Jeilongvirus murinae	BatPV-3	KJ641657	AIF74192.1
	Jeilongvirus rungweense	RulV	MW579602	QRN45791.1
	Lophuromys jeilongvirus 2	MMLV-2	MG573141	AVM86027.1
	Miniopteran jeilongvirus	ShaV	KY370098.1	AXR70620.1
	Myodes jeilongvirus	PMPV-1	MG516455	AVM86048.1
	Tailam jeilongvirus	TaiV	NC025355	AEU08865.1
Morbillivirus	Canine morbillivirus	CDV	AF014953.1	AAC26996.1
	Cetacean morbillivirus	DMV	AJ608288.1	CAE55659.1
	Feline morbillivirus	FeMV	JQ411014.1	AFH55518.1
	Measles morbillivirus	MeV	AB016162.1	BAA35122.1
	Phocine morbillivirus	PDV	KC802221.1	AGL33555.1
	Rinderpest morbillivirus	RPRV	X98291.3	CAA66935.2
	Small ruminant morbillivirus	RPV	AJ849636.2	CAH61259.1
Narmovirus	Mossman narmovirus	MossV	NC_005339.1	AAQ23993.1
	Myodes narmovirus	Bav-1	MF943130.1	ATW63190.1
	Nariva narmovirus	NarV	NC_017937.1	ACL97360.2
	Tupaia narmovirus	TupV	NC_002199.1	AAF63393.1
Respirovirus	Bovine respirovirus 3	BPIV-3	AF178654.1	AAF28259.1
	Caprine respirovirus 3	CPIV-3	NC_028362.1	AKO90683.1
	Human respirovirus 1	HPIV-1	AF457102.1	AAL89409.1
	Human respirovirus 3	HPIV-3	AB012132.1	ABY47607.1
	Murine respirovirus	MurV	MH557085.1	BAD74230.1
	Porcine respirovirus 1	PorV	JX857409.1	AGR39549.1
	Squirrel respirovirus	GSqV	LS992584	SYZ47181.1
Salemvirus	Salem salemvirus	SalV	NC_025386.1	AFM97198.1
Orthorubulavirus	Human orthorubulavirus 2	HPIV-2	X57559.1	CAA40788.1
	Human orthorubulavirus 4	HPIV-4	NC_021928.1	BAJ11747.1
	Mammalian orthorubulavirus 5	PIV-5	AF052755	AAC95518.1
	Mammalian orthorubulavirus 6	AlsV	MH972568	MH972568
	Mapuera orthorubulavirus	MapV	NC_009489.1	ABQ23938.1
	Mumps orthorubulavirus	MuV	AB040874.1	BAA94391.1
	Porcine orthorubulavirus	LPMV	BK005918	DAA06049.1
	Simian orthorubulavirus	SV-41	NC_006428.1	CAA45569.1
Pararubulavirus	Achimota pararubulavirus 1	AchV-1	JX051319.1	AFX75110.1
	Achimota pararubulavirus 2	AchV-2	JX051320.1	AFX75118.1
	Hervey pararubulaviru	HerV	KU672593	-
	Menangle pararubulavirus	MenV	JX112711.1	AFY09794.1

	Sosuga pararubulavirus	SoRV	KF774436.1	AHH02041.1
	Teviot pararubulavirus	TeV	KP271124.1	AJP33335.1
	Tioman pararubulavirus	TioV	AF298895.2	AAM82288.1
	Tuhoko pararubulavirus 1	TuhV-1	GU128080.1	ADI80715.1
	Tuhoko pararubulavirus 3	TuhV-3	GU128082.1	ADI80729.1
	Tuhoko pararubulavirus 2	TuhV-2	GU128081.1	ADI80722.1

Table S2. Nucleotide similarity (%) between our J paramyxovirus and other 14 species in genus *Jeilongvirus* based on a 709-bp fragment of the L gene.

L-709bp	BeiV	MisPV	BatPV-3	TaiV	ShaV	RoPV	BatPV-1	BatPV-2	MMLV-1	MMLV-2	FPaV	BeV	RulV	PMPV-1
**JPV**	70.6	65.5	62.4	71.7	73.2	63.4	64.8	63.4	65.0	68.6	67.2	62.9	62.2	70.8
**BeiV**	***	63.7	61.3	76.7	74.3	62.0	62.6	62.7	63.0	69.1	65.7	60.6	61.2	70.2
**MisPV**	***	***	65.3	64.3	64.8	74.8	75.1	74.4	67.4	65.0	66.1	63.6	66.0	63.0
**BatPV-3**	***	***	***	61.7	63.3	66.9	65.3	62.4	65.5	63.3	63.6	65.5	63.1	63.4
**TaiV**	***	***	***	***	72.9	62.4	64.4	64.1	67.9	71.0	67.8	62.9	62.3	72.7
**ShaV**	***	***	***	***	***	65.0	65.3	65.7	67.9	69.5	68.9	59.8	63.7	70.3
**RoPV**	***	***	***	***	***	***	74.7	76.0	65.8	66.2	67.5	63.1	64.3	63.4
**BatPV-1**	***	***	***	***	***	***	***	75.7	69.2	67.1	67.8	63.3	66.0	65.1
**BatPV-2**	***	***	***	***	***	***	***	***	66.1	66.2	66.7	64.3	63.7	62.9
**MMLV-1**	***	***	***	***	***	***	***	***	***	68.6	67.1	62.2	66.2	67.4
**MMLV-2**	***	***	***	***	***	***	***	***	***	***	67.7	63.6	65.8	69.1
**FPaV**	***	***	***	***	***	***	***	***	***	***	***	63.0	67.4	67.8
**BeV**	***	***	***	***	***	***	***	***	***	***	***	***	61.0	59.2
**RulV**	***	***	***	***	***	***	***	***	***	***	***	***	***	63.1

Table S3. Positive rate of J paramyxovirus in wild small mammals in six eco-climate regions in China.

**Wild Small Mammals**		**No. Positive/ No. Tested**						**No. Total Positive /No. Total Tested (%)**
**Family/Genus**	**Species**	**Northern**	**Northeastern**	**Inner Mongolia-Xinjiang**	**Central**	**Southern**	**Southwestern**	
Cricetidae		0/80	1/77	0/48	0/1		0/33	1/239 (0.42)
Allocricetulus	*Allocricetulus eversmanni*	0/6						0/6 (0)
Cricetulus	*Cricetulus barabensis*			0/2				0/2 (0)
	Cricetulus longicaudatus	0/28						0/28 (0)
	Cricetulus migratorius			0/41				0/41 (0)
Eothenomys	*Eothenomys cachinus*						0/1	0/1 (0)
	Eothenomys eleusis						0/8	0/8 (0)
	Eothenomys miletus						0/16	0/16 (0)
	Eothenomys proditor						0/8	0/8 (0)
Microtus	*Microtus fortis*		1/18		0/1			1/19 (5.26)
	Microtus oeconomus			0/1				0/1 (0)
	Microtus maximowiczii		0/1					0/1 (0)
Myodes	*Myodes rufocanus*	0/3	0/5					0/8 (0)

	Myodes rutilus		0/52					0/52 (0)
Phodopus	*Phodopus roborovskii*			0/4				0/4 (0)
Tscherskia	*Tscherskia triton*	0/43	0/1					0/44 (0)
Dipodidae				0/18				0/18 (0)
Allactaga	*Allactaga sibirica*			0/13				0/13 (0)
Dipus	*Dipus sagitta*			0/5				0/5 (0)
Muridae		15/1156	1/430	4/485	0/55	0/67	0/325	20/2518 (0.79)
Apodemus	*Apodemus agrarius*	1/220	1/170		0/31			2/421 (0.48)
	Apodemus chevrieri						0/56	0/56 (0)
	Apodemus draco	0/60						0/60 (0)
	Apodemus ilex						0/80	0/80 (0)
	Apodemus peninsulae	1/40	0/20					1/60 (1.67)
	Apodemus sylvaticus			0/15				0/15 (0)
Bandicota	*Bandicota indica*					0/14		0/14 (0)
Berylmys	*Berylmys bowersi*						0/1	0/1 (0)
Chiropodomys	*Chiropodomys gliroides*						0/1	0/1 (0)
Melomys	*Melomys burtoni*						0/2	0/2 (0)
Meriones	*Meriones libycus*			0/42				0/42 (0)
	Meriones meridianus			0/57				0/57 (0)
	Meriones tamariscinus			0/2				0/2 (0)
	Meriones unguiculatus			0/181				0/181 (0)
Micromys	*Micromys minutus*						0/1	0/1 (0)
Mus	*Mus musculus*	13/349	0/10	4/93				17/452 (3.76)
	Mus pahari						0/6	0/6 (0)
Niviventer	*Niviventer andersoni*	0/2					0/4	0/6 (0)
	Niviventer confucianus	0/24			0/9			0/33 (0)
	Niviventer coxingi						0/7	0/7 (0)
	Niviventer fulvescens				0/15			0/15 (0)
	Niviventer niviventer	0/18						0/18 (0)
Rattus	*Rattus andamanensis*					0/11		0/11 (0)
	Rattus brunneusculus						0/7	0/7 (0)
	Rattus norvegicus	0/158	0/230	0/20		0/37		0/445 (0)
	Rattus pyctoris			0/1				0/1 (0)
	Rattus steini						0/7	0/7 (0)
	Rattus tanezumi	0/285				0/5	0/144	0/434 (0)
	Rattus yunnanensis						0/9	0/9 (0)
Sciuridae		0/5	0/8	0/84			0/3	0/100 (0)
Callosciurus	*Callosciurus erythraeus*	0/5						0/5 (0)
Spermophilus	*Spermophilus dauricus*			0/28				0/28 (0)
	Spermophilus erythrogenys			0/31				0/31 (0)
	Spermophilus undulatus			0/25				0/25 (0)
Tamias	*Tamias sibiricus*		0/8					0/8 (0)
Tamiops	*Tamiops swinhoei*						0/3	0/3 (0)

Soricidae		0/65	0/3	0/2	0/16	0/18	0/44	0/148 (0)
Anourosorex	*Anourosorex squamipes*						0/15	0/15 (0)
Blarinella	*Blarinella quadraticauda*						0/4	0/4 (0)
Crocidura	*Crocidura horsfieldii*						0/1	0/1 (0)
	Crocidura lasiura	0/20			0/9		0/4	0/33 (0)
	Crocidura shantungensis	0/7			0/5			0/12 (0)
	Crocidura tanakae	0/21	0/2			0/1	0/5	0/29 (0)
Episoriculus	*Episoriculus caudatus*						0/3	0/3 (0)
Sorex	*Sorex bedfordiae*						0/8	0/8 (0)
	Sorex caecutiens				0/2			0/2 (0)
	Sorex isodon		0/1					0/1 (0)
Soriculus	*Episoriculus fumidus*	0/2						0/2 (0)
Suncus	*Suncus murinus*	0/15		0/2		0/17	0/4	0/38 (0)
Spalacidae				0/47				0/47 (0)
Myospalax	*Myospalax aspalax*			0/2				0/2 (0)
	Myospalax psilurus			0/45				0/45 (0)
Total		15/1306 (1.15)	2/518 (0.39)	4/684 (0.58)	0/72 (0)	0/85 (0)	0/407 (0)	21/3070 (0.68)
